# Naloxonazine, an Amastigote-Specific Compound, Affects *Leishmania* Parasites through Modulation of Host-Encoded Functions

**DOI:** 10.1371/journal.pntd.0005234

**Published:** 2016-12-30

**Authors:** Géraldine De Muylder, Benoit Vanhollebeke, Guy Caljon, Alan R. Wolfe, James McKerrow, Jean-Claude Dujardin

**Affiliations:** 1 Institute of Tropical Medicine, Department of Biomedical Sciences, Antwerp, Belgium; 2 University of California, San Francisco, San Francisco, CA, United States of America; 3 Laboratory of Molecular Parasitology, IBMM, Université Libre de Bruxelles, Gosselies, Belgium; 4 Department of Biomedical Sciences, Faculty of Pharmaceutical, Biomedical and Veterinary Sciences, University of Antwerp, Antwerp, Belgium; Ohio State University, UNITED STATES

## Abstract

Host-directed therapies (HDTs) constitute promising alternatives to traditional therapy that directly targets the pathogen but is often hampered by pathogen resistance. HDT could represent a new treatment strategy for leishmaniasis, a neglected tropical disease caused by the obligate intracellular parasite *Leishmania*. This protozoan develops exclusively within phagocytic cells, where infection relies on a complex molecular interplay potentially exploitable for drug targets. We previously identified naloxonazine, a compound specifically active against intracellular but not axenic *Leishmania donovani*. We evaluated here whether this compound could present a host cell-dependent mechanism of action. Microarray profiling of THP-1 macrophages treated with naloxonazine showed upregulation of vATPases, which was further linked to an increased volume of intracellular acidic vacuoles. Treatment of *Leishmania*-infected macrophages with the vATPase inhibitor concanamycin A abolished naloxonazine effects, functionally demonstrating that naloxonazine affects *Leishmania* amastigotes indirectly, through host cell vacuolar remodeling. These results validate amastigote-specific screening approaches as a powerful way to identify alternative host-encoded targets. Although the therapeutic value of naloxonazine itself is unproven, our results further demonstrate the importance of intracellular acidic compartments for host defense against *Leishmania*, highlighting the possibility of targeting this host cell compartment for anti-leishmanial therapy.

## Author Summary

Leishmaniasis is a poverty-related disease threatening 350 million people throughout the world. It is caused by the protozoan parasite *Leishmania*, a digenetic organism that switches from an extracellular stage in the sand fly vector to an intracellular stage in phagocytes of the vertebrate host. Drugs currently available to treat leishmaniasis are toxic to the patient and drug-resistant parasites are emerging, urging for new therapeutics. A novel strategy to tackle intracellular pathogens entails targeting the host cell, in order to indirectly interfere with pathogens growth. Here we analysed the mechanism of action of naloxonazine, a compound previously shown to specifically affect the intracellular amastigote stage of *Leishmania*. We show that this compound affects acidic compartments of macrophages and that these naloxonazine-induced modifications are responsible for *Leishmania* intracellular growth inhibition. Even though the therapeutic potential of naloxonazine itself is not proven, our results reveal the possibility of targeting host cell intracellular acidic compartments for anti-leishmanial therapy.

## Introduction

Protozoan parasites of the genus *Leishmania* are the causative agents of a wide variety of diseases ranging from self-healing or severe mucocutaneous lesions to a visceral disease which is lethal in the absence of treatment. Leishmaniasis is one of the most significant neglected tropical diseases, with an estimated 12 million people infected. *Leishmania* parasites have a digenetic life cycle; switching from an insect vector in which parasites dwell as extracellular promastigotes, to a mammalian host, where parasites reside exclusively intracellulary (intramacrophage amastigote stage).

Pentavalent antimonials (Sb^V^) like sodium stibogluconate (SSG) have been the first-line treatment against leishmaniasis for several decades but their clinical value has become compromised by increasing treatment failure and the emergence of resistant parasites. This concern is particularly important in the Indian subcontinent where visceral leishmaniasis (VL) caused by *Leishmania donovani* is endemic and where most VL cases occur [1]. Current treatment alternatives consist of amphotericin B, miltefosine or paromomycin (in mono- or combination therapy) but these compounds also have drawbacks including cost, toxicity or decreased efficacy after a few years of use [2].

Although the mechanism of action of these compounds is not fully understood, they are all known to target *Leishmania* components, therefore directly interfering with parasite growth: amphotericin B forms a complex with ergosterol, the main sterol of *Leishmania* cellular membrane, leading to formation of aqueous pores and increased membrane permeability [3]; miltefosine has been shown to inhibit the parasite cytochrome c oxidase and to cause apoptosis-like processes *in L*. *donovani* [4]; and paromomycin is an aminoglycoside antibiotic that inhibits protein synthesis in *Leishmania* with low host cell toxicity [5]. Sb^V^ on the other hand, has been shown to target both the parasite and the host cell: Sb^V^ is reduced to trivalent antimony (Sb^III^), which directly alters the parasite redox metabolism and antioxidant defense system, but Sb^V^ itself also indirectly affects parasite survival by increasing host cell production of toxic oxygen and nitrogen intermediates, thereby creating additional oxidative and nitrosative stress upon Sb^III^-sensitized parasites [6]. Antimonial anti-leishmanial activity is thus partly indirect, targeting host cell pathway(s) that consequently affect *Leishmania* intracellular development.

Targeting host cell pathways to interfere with the intracellular development of pathogens is a strategy increasingly investigated for antimicrobial therapy that might bring novel therapeutic approaches in a context of increased treatment failure and poor alternatives [7,8]. Following this line, a recent high-throughput screening campaign against kinetoplastids at GlaxoSmithKline identified several compounds associated with human proteins with no known homologs in kinetoplastids, highlighting the possibility of targeting host-pathogen interactions[9].

Here we report the host-dependent anti-leishmanial activity of naloxonazine, a mu-opioid receptor (MOR) antagonist. This compound was first identified in a high-throughput screen against *Leishmania donovani* intracellular amastigotes [10]. We now show that it affects host cell intracellular compartments thereby inhibiting *Leishmania* establishment in the phagolysosomal vacuole.

## Methods

### Parasite strains, culture conditions and compounds

Parasite strains used in this study included *L*. *donovani* 1S2D (MHOM/SD/62/1S-cl2D), *L*. *donovani* 1S2D expressing the enhanced green fluorescent protein (eGFP) and two *L*. *donovani* clones of clinical isolates from the Terai endemic region in Nepal (MHOM/NP/02/BPK282/0cl4 and MHOM/NP/03/BPK275/0cl18 respectively susceptible and resistant to SSG and further designated SSG-S BPK282 and SSG-R BPK275). Promastigotes were maintained at 26°C in hemoflagellate modified Eagles’s medium (HOMEM) supplemented with 20% Foetal Bovine Serum (FBS). Differentiation of promastigotes into axenic amastigotes was achieved as described previously [11]. THP-1 cells (human acute monocytic leukemia cell line–ATCC TIB202) were grown in RPMI supplemented with 10% FBS and 50 μM 2-mercaptoethanol at 37°C in 5% CO_2_. For *Leishmania* infections, THP-1 cells were treated with 0.1 μM phorbol myristate acetate (PMA, Sigma) at 37°C for 48 h to achieve differentiation into adherent, non-dividing macrophages. Cells were washed and incubated with complete RPMI medium containing stationary phase *L*. *donovani* promastigotes at a macrophage/promastigote ratio of 1/10. After 4 h incubation at 37°C, non-internalized promastigotes were removed by 3 successive washes with PBS and incubated with naloxonazine, naloxone, β-funaltrexamine, CTOP, endomorphine, DAMGO, sinomenine, concanamycin A (all purchased from Sigma) or imatinib (Cell Signaling Technology) for 24 to 72 h. Half maximal inhibitory concentrations (GI_50_) were determined using a high-content imaging assay as described previously [10]. Briefly, compounds were serially diluted 3-fold in DMSO, with final assay concentrations ranging from 50 μM to 0.02 μM (1% final concentration of DMSO), 2 μM amphotericin B and 1% DMSO were used as positive and negative controls respectively. For confocal microscopy, infected cells were washed with PBS, fixed for 30 minutes with 4% formaldehyde, rinsed again with PBS and stained with 4’,6’-diamidino-2-phenylindole (DAPI 300 nM). Images were acquired with an LSM 700 Zeiss confocal microscope.

### Evaluation of naloxonazine stability by LC-MS/MS

20 μM naloxonazine was incubated for 50 h in RPMI 10% FBS- 50 μM 2-mercaptoethanol with or without THP-1 cells at a concentration of 10^6^ cells/ml. 100 μl of culture media were collected at different time points (T0; 0,5; 1; 5; 10; 20; 30; 45 and 50 h) and kept frozen. 20 μl of these samples were then mixed with 40 μl cold acetonitrile containing either 2 μM naloxone or 2 μg/mL K777 (*N*-methylpiperazine-PhehomoPhe-vinylsulfone-phenyl), centrifuged, and 3 μL per sample injected into an API4000 (AB Sciex) LC-MS/MS system and analysed with positive-ion-mode electrospray ionization. A binary mobile phase (A,15% methanol:water; B, 100% methanol:water; both containing 0.1% formic acid, 0.1% ACN and 160 mg/L NH_4_OAc) was pumped at 0.5 mL/min through a 4.6 x 50 mm, 5 μm, 100 Å pore Kinetex C18 column (Phenomenex). The gradient used was: 0–0.5 min, 0% B; 0.5–3.0 min, linear ramp to 100% B; 3.0–4.0 min, 100% B; 4.0–4.5 min, linear ramp to 0% B; 4.5–7.0 min, 0% B. MS settings were as follows: common settings were temperature = 600°C, GS1 (ion source nebulizer gas) = GS2 (ion source heater gas) = 50 lbf in^-2^; CUR (curtain gas) = 35 lbf in^-2^; CAD (collision gas) = 12 lbf in^-2^; IS (ion spray voltage) = 5500 V; analyte-specific settings for naloxonazine, naloxone and K777, repectively, were DP (declustering potential) = 101 V, 76 V and 56 V; EP (entrance potential) = 13.2 V, 10 V and 10 V; CE (collision energy) = 47 eV, 37 eV and 57 eV; CXP (collision cell exit potential) = 18 V, 14 V and 18 V. The MS/MS transitions used were naloxonazine, m/z 651.5 → 325.3; naloxone, 328.3 → 253.1; K77, 575.5 → 101.3, and retentions were 3.16, 3.03 and 4.43 min, respectively.

### siRNA-mediated MOR knock-down

MOR-specific siRNA was purchased from Qiagen (Hs_OPRM1_7 FlexiTube siRNA). THP-1 cells were transfected with 1 μM of siRNA using the Amaxa nucleofector kit V following the manufacturer’s instructions. Control cells were mock transfected in parallel (“mock” control). After nucleofection, THP-1 cells were resuspended in complete RPMI medium (5. 10^5^ cells/ml), treated with PMA for 24 h and infected with stationary phase *L*. *donovani* 1S2D promastigotes as described above. Parasite infectivity was assessed 48 h after infection. Down-regulation of MOR mRNA level was analysed 24 h after nucleofection and 48 h after infection by qRT PCR as described below using the following primer sets: *MOR* fwd: 5’ GGTACTGGGAAAACCTGCTGAAGATCT, rev: 5’ GGTCTCTAGTGTTCTGACGAATTCGAGTGG and *18S rRNA*: Fwd 5' ACCGATTGGATGGTTTAGTGAG, Rev 5' CCTACGGAAACCTTGTTACGAC. The relative expression level of MOR was determined based on the Ct value normalized to the Ct value of the reference 18S rRNA. siRNA treated cells were compared to the mock transfected cells.

### RNA extraction and microarray

Non-infected, PMA-activated THP-1 cells (5.10^5^ cells/ml, 10 ml) were treated with 10 μM of naloxonazine or 10 μM of naloxone. After 4 h of treatment, compounds were removed by 2 washes with PBS and cells were further incubated 20 h in compound-free RPMI medium. This time-point was chosen to maximize the chances of detecting naloxonazine-induced transcriptional changes while limiting the observation of downstream effects. Total RNA was extracted using TRIzol (Invitrogen) and amplified with the Amino Allyl MessageAmp™ II aRNA Amplification Kit (Ambion) following the manufacturer’s protocol. The monofunctional NHS-ester Cy3 and Cy5 dyes (GE Healthcare Life Sciences) were coupled with 10 μg amplified RNA. The two aRNA pools to be compared were mixed and applied to the Human Exonic Evidence Based Oligonucleotide (HEEBO) array (Stanford Functional Genomics Facility). HEEBO oligonucleotide set consists of 44,544 70mer probes that were designed using a transcriptome-based annotation of exonic structure for genomic loci. Four samples (two from naloxonazine-treated and two from naloxone-treated cells) were competitively hybridized on two individual chips (further called “array1” and “array2”). The hybridization was performed at 63°C for 16 h in a humidified slide chamber containing the labeled probe, 3X SSC, and 0.2% SDS. After hybridization, the hybridization chamber was removed from the 63°C water bath, washed with 0.6X SSC, 0.03% SDS, and then 0.06X SSC. Microarrays were scanned using a GenePix Pro Axon 4000B scanner, data were analysed with the Acuity software (Molecular Devices). Fluorescent data were background adjusted and the ratios of naloxonazine-treated to naloxone-treated data were calculated for each probe set. Sets of genes showing a ratio > 2 were functionally clustered using DAVID [12,13].

### qRT-PCR

RNA was extracted as described above, from non-infected THP-1 cells treated with 10 μM of naloxonazine for 4 h and further incubated 20 h in compound-free RPMI medium. cDNA synthesis was done with Transcriptor Reverse Transcriptase (Roche) and a 15-mer oligo(dT) primer from 1 μg of total RNA. qPCRs were run with the SensiMix SYBR no-ROX kit (Bioline) on a LightCycler 480 (Roche). The following primer sets were used: *vATPase subunit c (ATP6V0C)*: Fwd 5' ATGTCCGAGTCCAAGAGC, Rev 5' CTACTTTGTGGAGAGGATGAG; *vATPase subunit a (TCIRG1)*: Fwd 5’ ATCTGGCAGACTTTCTTCAG, Rev 5’ AAGATGCTGGTGGCGCGACT; B-*Actin (ACTB)*: Fwd 5' TCCCTGGAGAAGAGCTACGA, Rev 5' AGCACTGTGTTGGCGTACAG; *18S rRNA*: Fwd 5' ACCGATTGGATGGTTTAGTGAG, Rev 5' CCTACGGAAACCTTGTTACGAC. The relative expression levels of vATPase and actin were determined based on the Ct value of each gene normalized to the Ct value of the reference 18S rRNA. Naloxonazine treated cells were compared to untreated cells.

### Western blotting

Total protein extracts of THP-1 cells infected with *L*.*d*. 1S2D, treated or not with 10 μM of naloxonazine for 24 or 48 h, were prepared in Laemmli sample buffer (Bio Rad) and analysed by SDS-PAGE and western blotting. The equivalent of 10^5^ cells were loaded per well. Membranes were first incubated with an anti-vATPase subunit a3 (rabbit polyclonal anti-TCIRG1, abcam, 1:1000), and an anti-rabbit HRP (1:5000), then stripped with Restore western blot stripping buffer (Thermofisher) and further incubated with an anti α-tubulin (mouse monoclonal, abcam, 1:1000) and an anti-mouse HRP (1:5000). Proteins were detected by chemoluminescence following the manufacturer’s instructions (PierceECL western blotting substrate, Thermofisher). Quantitative densitometry was performed using Image J.

### Lysotracker staining

THP-1 cells infected with eGFP-expressing *L*. *donovani* were treated or not with 10 μM of naloxonazine, 10 μM of naloxone or 80 nM of concanamycin A for 24 h, or co-treated with either naloxonazine (10 μM) and concanamycin A (80 nM) or naloxone (10 μM) and concanamycin A (80 nM) for 24 h, then stained with 180 nM of the Lysotracker red DND-99 (Life Technologies) for 1 h at 37°C. For microscopy, cells were further stained with 500 nM of the nucleic acid stain Hoechst 33342 (Life Technologies) and images were acquired with an LSM 700 Zeiss confocal microscope. For flow cytometry, Lysotracker red DND-99 stained cells were first trypsinised with TrypLE Select (Invitrogen), washed with PBS and analysed with a BD FACSVerse flow cytometer and the BD FACSSuite software.

### Statistical analysis

GraphPad Prism 5 software was used to determine the statistical significance (Two-way ANOVA or t-test as specified in the figure legends).

### Ethics statement

Clinical samples were from an already existing collection (B.P. Koirala Institute of Health Sciences in Dharan). All samples were anonymized and their use was approved by the review boards of the Nepal Health Research Council, Kathmandu, the Institute of Tropical Medicine, Antwerp and the Antwerp University.

## Results

### 1- Naloxonazine anti-leishmanial activity is dependent on the presence of the host cell and affects both SSG-R and SSG-S parasites

**Fig 1:**
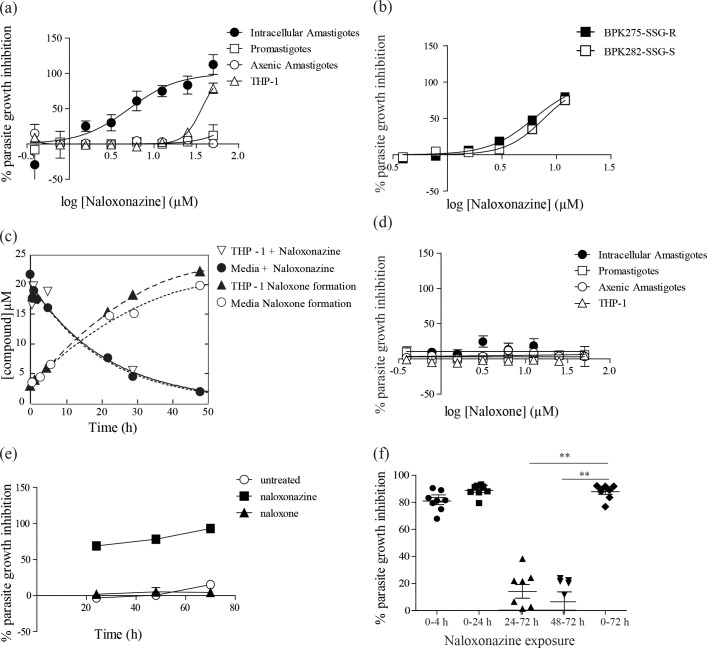
Effect of naloxonazine on *Leishmania* intracellular growth. Dose response curve for naloxonazine (a) and naloxone (d) against *L*. *donovani* 1S2D intracellular amastigotes, promastigotes, axenic amastigotes and the THP-1 host cell; and dose response curve for naloxonazine against intracellular amastigotes of *L*. *donovani* clinical isolates SSG-R or SSG-S (b). Disappearance of naloxonazine and appearance of naloxone during incubation with or without the THP-1 host cell (c). Effect of naloxonazine (10 μM) and naloxone (10 μM) on *L*. *donovani* 1S2D intracellular amastigotes growth after 24, 48 and 70 h (e). Data are mean ± SD of three independent experiments. Exposure of *L*. *d*. 1S2D-infected THP-1 cells to naloxonazine (10 μM) for different timeframes: 4, 24 or 72 hours (respectively labeled 0–4; 0–24; 0–72) or addition of naloxonazine (10 μM) 24 or 48 hours after infection (labeled 24–72 and 48–72), parasite infectivity was assessed after 72h. Data are pooled from two independent experiments, **p < 0,01 unpaired t-test comparing each condition to the 72 h incubation with naloxonazine (f).

The activity of naloxonazine was tested *in vitro* against three stages of *Leishmania donovani*: insect-stage promastigotes, intracellular amastigotes (within the macrophage host cell) and host cell-free axenic amastigotes (an amastigote-like stage obtained from differentiation of promastigotes *in vitro* in the absence of a host cell). Naloxonazine was shown to be active against the intracellular amastigote stage with a half maximal inhibitory concentration (GI_50_) of 3,45 μM. It exhibited a reasonable selectivity, with a GI_50_ of 34 μM against the THP-1 host cell. Remarkably, the compound was inactive against *L*. *donovani* promastigotes or axenic amastigotes, indicating the importance of the host cell microenvironment for compound activity (Fig 1A). Naloxonazine was also tested against two *L*. *donovani* clinical isolates, one showing susceptibility, the other showing resistance to antimonials (SSG-S and SSG-R strains). The activity of naloxonazine against these isolates was comparable, suggesting that the mechanism of resistance developed against SSG does not affect naloxonazine activity (Fig 1B).

### 2- Naloxonazine but not naloxone inhibits parasite intracellular growth

The necessity of the host cell presence for naloxonazine’s anti-leishmanial activity might be hypothesized to be linked to the metabolic properties of macrophages, i.e. naloxonazine could be a prodrug dependent on host cell metabolism to gain anti-leishmanial activity. In order to define the exact chemical moiety endowed with anti-leishmanial activity and to evaluate whether the macrophage would metabolize naloxonazine into an active compound, naloxonazine stability during incubation in THP-1 cell medium was evaluated by LC-MS/MS in the presence or absence of THP-1 host cells. Naloxonazine had a half-life of 15 h and was shown to be degraded into naloxone, another MOR antagonist, regardless of the presence of THP-1 macrophages (Fig 1C). Interestingly, naloxone was shown to be inactive against all stages of *L*. *donovani*, including the intracellular amastigotes (Fig 1D). Naloxonazine is thus not a prodrug activated by the macrophage host cell but its activity seems inherent, associated with its unperturbed chemical identity.

### 3- Naloxonazine is active at early stages of infection

The kinetics of naloxonazine activity showed that parasite growth was already inhibited by 70% after 24 h of compound incubation; 95% of growth inhibition was achieved after 72 h incubation (Fig 1E). Remarkably, exposure of infected macrophages to naloxonazine for 4 h, followed by a washing step to remove the compound from the cells and an additional incubation of 70 h, led to the same level of parasite growth inhibition as a 72 h-incubation with the compound (Fig 1F). This observation is in accordance with the degradation time of naloxonazine (Fig 1C). Moreover, delaying addition of naloxonazine to 24 or 48 h after infection reduced its anti-leishmanial effect by 75%, suggesting that naloxonazine is most active at early stages of infection.

### 4- Macrophage MOR are not involved in parasite intracellular growth

**Fig 2:**
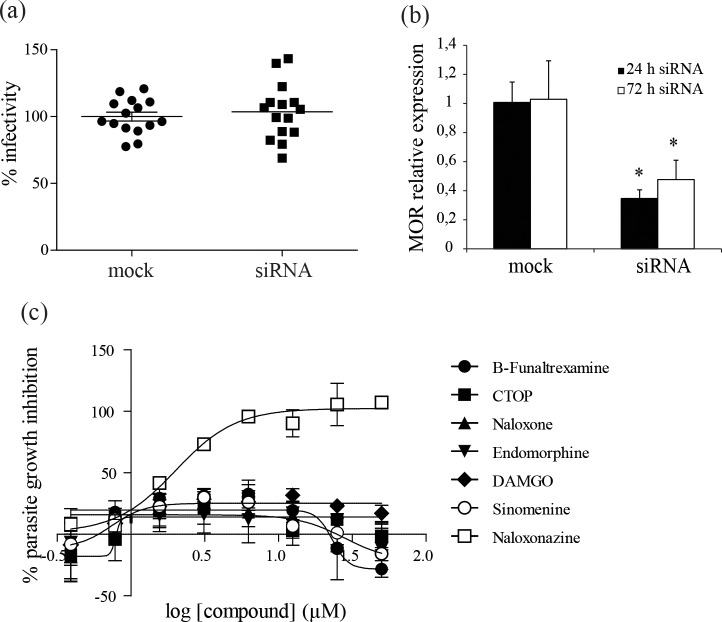
Importance of opioid receptors for *L*. *donovani* intracellular growth. *L*.*donovani* infection of THP-1 cells transfected with MOR-targeting siRNA, infectivity was normalized to mock-transfected controls. Data are pooled from three independent experiments (a). Expression level of MOR was assessed in siRNA-transfected compared to mock-transfected THP-1 cells, 24 h after transfection and 48 h after *L*. *donovani* infection (i.e. 72 h after transfection). Data are mean ± SD of three independent experiments, *p < 0,05 two-way ANOVA comparing siRNA-transfected and mock-transfected THP-1 cells (b). Dose response for MOR antagonists naloxonazine, β-funaltrexamine and CTOP, and MOR agonists endomorphine, DAMGO and sinemonine against *L*. *donovani* 1S2D intracellular amastigotes. Data are mean ± SD of three independent experiments (c).

We hypothesized that naloxonazine anti-leishmanial activity is dependent on its antagonistic effect towards MOR of macrophages. siRNA-mediated knock-down of MOR was therefore carried out in THP-1 cells to evaluate the importance of these receptors for parasite intracellular growth. Fifty percent down-regulation of MOR mRNA was obtained, but this was not accompanied by changes in infection levels (Fig 2A and 2B). The amount or function of the MOR protein was not assessed in the siRNA-treated cells; however, phenocopy of naloxonazine’s effect on *L*. *donovani* growth could not be observed with a set of other antagonists or agonists of opioid receptors (Fig 2C), supporting the conclusion that the activity of naloxonazine on *Leishmania* intracellular growth is independent of opioid receptors.

### 5- Naloxonazine affects acidic compartments of the host cell which in turn limit *L*. *donovani* intracellular growth

**Fig 3:**
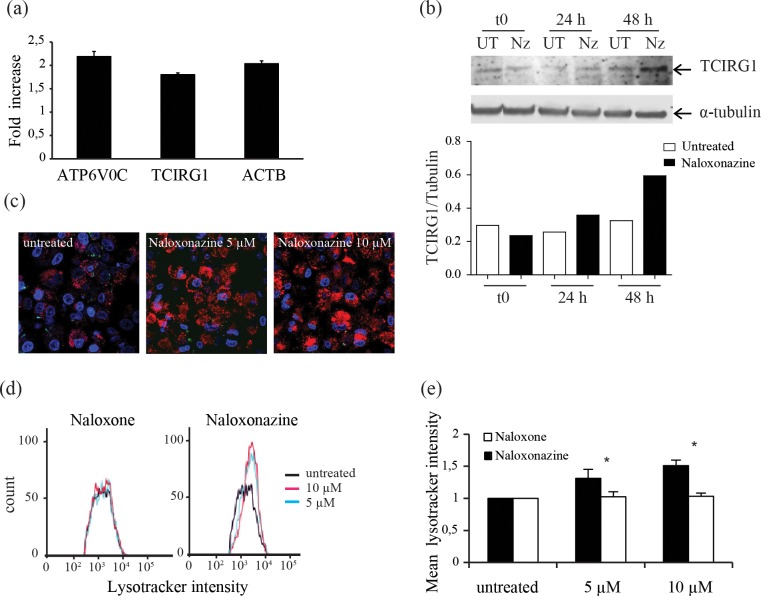
Effect of naloxonazine on host cell intracellular acidic compartments. Expression level of vATPase (ATP6V0C and TCIRG1 subunits) and actin (ACTB) after naloxonazine treatment was measured by qRT-PCR on infected THP-1 cells; data are mean ± SD of triplicates from one representative of two experiments (a). Protein levels of vATPase subunit a3 (TCIRG1) in infected macrophages treated with naloxonazine (Nz) for 24 or 48 h (UT = untreated). Protein levels were quantified by densitometry and normalized to the α-tubulin loading control (b). THP-1 cells infected with eGFP-tagged *L*. *donovani* (green) treated or not with naloxonazine and stained with Lysotracker (red) and Hoechst 33342 (blue) were analysed by confocal microscopy (c) and flow cytometry (d, e). Data are mean ± SD of two independent experiments each performed with biological triplicates, * p<0,05 two-way ANOVA comparing naloxonazine and naloxone treated cells.

Microarray profiling of THP-1 cells treated with naloxonazine or naloxone was performed to pinpoint host cell pathways differentially affected by the drugs and identify pathways that could be important for *Leishmania* intracellular growth. A 4 h-compound incubation followed by an additional compound-free incubation of 20 h was chosen, to maximize the chances of detecting naloxonazine-induced transcriptional changes while limiting the observation of downstream effects. Two percent of the probes showed at least two-fold differential gene expression in naloxonazine versus naloxone treated THP-1 cells. These upregulated genes were functionally clustered with the Database for Annotation, Visualization and Integrated Discovery (DAVID [12]). Both the vacuolar H+ ATPase gene family and actin and actin-related genes clusters were perturbed, pointing to a possible effect of naloxonazine on phagolysosome formation or maturation (Table 1). The expression level of two vATPase subunits and actin was also analysed by qRT PCR in naloxonazine-treated compared to untreated cells at the same time point as the one chosen for the microarray experiment. Upregulation of these genes after naloxonazine treatment was confirmed (Fig 3A). Upregulation of vATPase subunit a3 was also established at protein level after 24 and 48 h of treatment (Fig 3B). To test whether naloxonazine could affect the phagolysosome, *L*. *donovani*-infected THP-1 cells treated or not with naloxonazine were stained with Lysotracker, a fluorescent acidotropic probe that accumulates in cellular compartments with low internal pH. Stained cells were analysed by confocal microscopy and flow cytometry. The intensity of the Lysotracker signal was increased after naloxonazine treatment, indicating an increased combined volume of acidic vacuoles (Fig 3C, 3D and 3E). These results suggested that naloxonazine influenced the intracellular acidic compartments of the host cell.

**Table 1:** Vacuolar H+ ATPase gene family members and actin-related genes upregulated in naloxonazine-treated cells.

genes	name	fold change array1	fold changearray2
**ATP6V1G2**	ATPase, H+ transporting, lysosomal 13kDa, V1 subunit G2	2,03	2,14
**ATP6AP1**	ATPase, H+ transporting, lysosomal accessory protein 1	2,02	1,84
**ATP6V0D1**	ATPase, H+ transporting, lysosomal 38kDa, V0 subunit d1	2,09	2,28
**ATP6V0C**	ATPase, H+ transporting, lysosomal 16kDa, V0 subunit c	1,72	2,25
**ATP6V0A4**	ATPase, H+ transporting, lysosomal V0 subunit a4	2,11	2,55
**ATP6V1F**	ATPase, H+ transporting, lysosomal 14kDa, V1 subunit F	1,79	2,16
**ATP6V1C2**	ATPase, H+ transporting, lysosomal 42kDa, V1 subunit C2	1,62	2,7
**ATP9B**	ATPase, Class II, type 9B	2,46	2,87
**ATP5E**	ATP synthase, H+ transporting, mitochondrial F1 complex	2,15	2,05
**ATP5G2**	ATP synthase, H+ transporting, mitochondrial F0 complex	1,93	2
**ABCC12**	ATP-binding cassette, sub-family C (CFTR/MRP), member 12	2,01	1,98
**TAPBP**	TAP binding protein (tapasin)	2,07	2,45
**TCIRG1**	T-cell, immune regulator 1, ATPase, H+ transporting, lysosomal	2,15	2,52
**ACTB**	actin, beta	3,1	3,23
**ARPC1B**	actin related protein 2/3 complex, subunit 1B, 41kDa	2,14	2,23
**ARPC2**	actin related protein 2/3 complex, subunit 2, 34kDa	1,65	2,27
**CAPZB**	capping protein (actin filament) muscle Z-line, beta	2,14	2,75
**CAPZA1**	capping protein (actin filament) muscle Z-line, alpha 1	2,27	2,65
**TMSB10**	thymosin, beta 10	1,76	2,24
**TMSB4**	thymosin, beta 4	1,6	2,1

**Fig 4:**
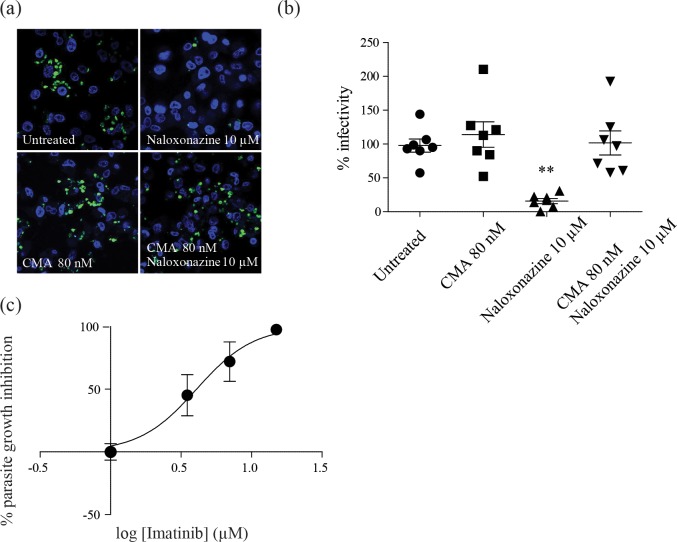
Host cell acidic compartments affect *L*. *donovani* intracellular growth. THP-1 cells infected with eGFP-tagged *L*. *donovani* (green) treated with 10 μM naloxonazine or 80 nM concanamycin A (CMA) or co-treated with both naloxonazine (10 μM) and CMA (80 nM) were stained with DAPI (blue) and analysed by confocal microscopy (a). Infectivity was estimated by manual counting and normalized to untreated cells, **p<0,01 unpaired t-test comparing treated and untreated cells (b). Dose response for imatinib against *L*. *donovani* 1S2D intracellular amastigotes (c). Data are representative of three independent experiments.

In order to determine if naloxonazine-induced changes in intracellular compartments were responsible for the effect on *L*. *donovani* intracellular growth, *L*. *donovani*-infected THP-1 cells were treated with both naloxonazine and the vATPase inhibitor concanamycin A. Remarkably, concanamycin A was sufficient to restore normal infection levels in naloxonazine-treated cells, confirming the importance of host cell-acidic compartments for controlling *L*. *donovani* intracellular growth (Fig 4A and 4B). To further establish the importance of acidic compartments for *Leishmania* intracellular growth inhibition, we evaluated the activity of imatinib, an inhibitor of Abelson tyrosine kinase previously shown to trigger intracellular acidification in monocyte-derived macrophages [14, 23]. In agreement with our previous observations, imatinib exhibited anti-leishmanial activity in the low micromolar range (GI_50_ of 4 μM; Fig 4C).

## Discussion

We showed that naloxonazine does not directly target *L*. *donovani* but rather interferes with intracellular acidic compartments of the host cell. Upon infection, *Leishmania* parasites are recognized by phagocytic cells and internalized by phagocytosis in a phagosome/parasitophorus vacuole. During the process of phagocytosis, the phagosome matures through fusion with endosomes and lysosomes, ultimately leading to a highly microbicidal environment. One component of this microbicidal response is the acidification of the phagosome due to the recruitment of the vATPase proton pump to the mature phagosomal membrane [15]. Intracellular pathogens can survive these extreme conditions by arresting phagosomal maturation at an early non-microbicidal stage, developing resistance to the microbicidal arsenal of the phagolysosome, or escaping from the phagosome into the cytosol.

It is well established that *Leishmania* amastigotes are adapted to the acidic pH found in the parasitophorus vacuole and are able to proliferate under these conditions [16,17]. In contrast, it has been proposed that promastigotes, the parasite stage of the insect vector, delay phagosome maturation to avoid destruction before differentiation into amastigotes [18,19]. Although this hypothesis has been challenged by the observation that promastigote-containing parasitophorus vacuoles do fuse with lysosomes [20], the importance of acidic pH for controlling intracellular *Leishmania* growth is well recognized. Infection studies in Stat1 deficient mice for instance showed an increased *Leishmania* intracellular growth that was associated with an increase in phagosomal pH [21].

Our study demonstrated a naloxonazine-induced increased expression of the vATPase transporter as well as an increase of Lysotracker-positive intracellular compartments which was associated to an enhanced capacity of the host cell to control infection. Whether naloxonazine influences the pH of the parasitophorus vacuole or the amount of acidic vacuoles is unclear at this stage. Time-course analysis supported a naloxonazine anti-leishmanial effect at early stages of infection, in accordance with previous observations showing the importance of acidic compartments in the early steps of infection [18, 19]. The microarray analysis performed in this study also showed upregulation of actin and some actin-related genes in naloxonazine-treated cells. However, further investigation is required to assess the importance of actin for the anti-leishmanial activity of naloxonazine.

The molecular pathways affected by naloxonazine that lead to modification of the phagosome are yet to be determined. Naloxonazine is a MOR antagonist [22]; however, albeit such receptors are expressed by macrophages and more specifically by the THP-1 cell line, they could not be linked to *Leishmania* growth inhibition. Naloxone, β-funaltrexamine or CTOP, other MOR antagonists, were inactive against *L*. *donovani* and knock-down of MOR in THP-1 cells did not affect *L*. *donovani* intracellular growth. Moreover, naloxonazine is a very potent MOR antagonist (Kd < 2 nM) while its activity against *L*. *donovani* is in the micromolar range. These data suggest that the cellular target of naloxonazine in this case is independent of MOR.

Targeting phagolysosome acidification to fight against intracellular pathogens is a strategy that has previously been validated for *Mycobacterium tuberculosis*, a pathogen that infects macrophages through delayed phagosome maturation. Imatinib, an Abelson tyrosine kinase inhibitor used to treat early chronic myeloid leukemia, was shown to decrease the pH of intracellular compartments which in turn reduced *M*. *tuberculosis* intracellular growth *in vitro* and *in vivo* (14,23). Whether Abelson tyrosine kinases are involved in the naloxonazine-induced pH decrease of intracellular compartments deserves further investigation.

Host-directed therapies (HDT) are considered an innovative strategy for infectious diseases, given the concern over parasites evolving resistance to current treatments, combined with the recognition of the importance of host determinants for progression of infections. HDTs are receiving increasing attention in treatment of tuberculosis for instance, and are expected to improve treatment outcomes against drug-susceptible as well as multi-drug-resistant strains [24]. Examples of HDTs under evaluation for tuberculosis treatment include anti-inflammatory compounds, statins and imatinib [25]. In antiviral therapy, HDTs have also been raised as interesting alternatives and possible solutions for limiting the emergence of drug resistance [26].

Treatment against leishmaniasis is also jeopardized by increasing treatment failure and drug resistance. Of importance, treatment failure does not necessarily correlate with parasite drug resistance (at least against SSG or miltefosine), highlighting the importance of the host background for treatment outcome in this case [27]. This observation would therefore also argue in favor of HDTs against leishmaniasis. In this context, immunotherapy has received considerable interest in recent years, with the idea of modulating the immune system to achieve a protective response and parasite elimination [28,29]. Combination of immuno- and chemo-therapy is believed to be synergistic, allowing infection control while reducing the threat of drug resistance.

Whether HDTs would be less likely to induce resistance remains an interesting and important question, and for *Leishmania* at least, the example of Sb^V^ raises concern. Indeed, although Sb^V^ targets host cell defense pathways, Sb^V^-R parasites have been isolated in the Indian subcontinent, and notably these parasites showed increased infectivity [30]. This ought to raise concern of possibly generating more virulent strains should parasites become resistant to immuno-therapy.

Targeting host-encoded functions unrelated to the immune response but important for parasite invasion or intracellular development provides additional options for HDTs for leishmaniasis and intracellular pathogens in general. This is exemplified by naloxonazine or imatinib and their interference with endocytic components of the host cell.

Although the potential of naloxonazine itself as a therapeutic anti-leishmanial drug is unproven, targeting pathways linked to phagosome acidification remains of great interest. In addition, naloxonazine was as potent against both SSG-R and SSG-S clinical isolates, highlighting the possible benefits of such a drug target for parasites resistant to classic chemotherapy.
